# Quasistatic transfer protocols for atomtronic superfluid circuits

**DOI:** 10.1038/s41598-021-82386-y

**Published:** 2021-02-04

**Authors:** Yehoshua Winsten, Doron Cohen

**Affiliations:** Department of Physics, Ben-Gurion University of the Negev, 84105 Beer-Sheva, Israel

**Keywords:** Physics, Quantum physics, Statistical physics, thermodynamics and nonlinear dynamics

## Abstract

Quasi-static protocols for systems that feature a mixed phase-space with both chaos and quasi-regular regions are beyond the standard paradigm of adiabatic processes. We focus on many-body system of atoms that are described by the Bose–Hubbard Hamiltonian, specifically a circuit that consists of bosonic sites. We consider a sweep process: slow variation of the rotation frequency of the device (time dependent Sagnac phase). The parametric variation of phase-space topology implies that the quasi-static limit is not compatible with linear response theory. Detailed analysis is essential in order to determine the outcome of such transfer protocol, and its efficiency.

## Introduction

Considering a closed Hamiltonian driven system, such as a particle in a box with moving wall (aka the piston paradigm), the common claim in Statistical Mechanics textbooks is that quasi-static (QS) processes are adiabatic, with vanishing dissipation in this limit, which implies thermodynamic reversibility. Indeed this claim can be established for an *integrable* system by recognizing that the action-variables are adiabatic invariants^1^. Also the other extreme, of a slowly driven completely *chaotic* system, has been addressed^2–4^, leading to the mesoscopic version of the Kubo linear-response result and the associated fluctuation-dissipation phenomenology^5–8^. But generic systems are neither integrable nor completely chaotic. Rather they have *mixed phase space*. For such system the adiabatic picture fails miserly^9–12^, because the variation of the control parameter is associated with structural changes in phase space topology: tori merge into chaos, and new sets of tori are formed later on. This can be regarded as the higher-dimensional version of separatrix crossing^13–23^, where the so-called Kruskal–Neishtadt–Henrard theorem is followed.

In the present work we consider the implications of having mixed phase space with regard to quasi-static transfer protocols (QSTP). Specifically we focus on Bose–Hubbard circuits , and ask what is the outcome of a QS process whose aim is to transfer particles coherently from one orbital to another orbital. Systems that are described by the Bose–Hubbard Hamiltonian (BHH) are of major interest both theoretically and experimentally^24–27^. The simplest configuration is the BHH dimer (two sites), aka the Bosonic Josephson Junction (BJJ), see^28^ and references therein. More generally there is an interest in lattice ring circuits that can serve as a SQUID or as a useful Qubit device^29–32^. The hope is that coherent operation might be feasible for BHH configuration with few sites, as already established for protocols that involve two sites (BJJ). The most promising configuration is naturally the 3-site trimer^33–51^. For the analysis of such circuit one has to confront the handling of an underlying mixed phase space^49,50,52^. In particular the implications of mixed phase-space on the stability of superflow has been explored in Ref.^49–51^.

Striking forms of irreversibility can be observed in hysteresis experiments with ultracold atoms, both is double well geometry^58^ and in ring geometry^59,60^. For related theoretical studies see for example^53–57^, where the emphasis is mainly on the parametric bifurcations of fixed points in phase space (notably the so-called swallow-tail loops). More recently the effect of *chaos* has been taken into account while studying the efficiency of a nonlinear stimulated Raman adiabatic passage^11^; and the Hamiltonian hysteresis that follows the reversal of the driving scheme^12^.

Our interest in QSTP is motivated by hysteresis experiments with atomtronic superfluid circuits, as in^60^. Namely, we consider the following protocol for a ring-shaped circuit: (1) Initially, at the preparation stage, all the particles are condensed into the lowest momentum orbital that has a zero winding number; (2) The rotation frequency $\Phi$ of the ring is gradually changed, aka sweep process; (3) The final state of the system is probed, and the momentum distribution is measured. One possibility would be to find that all the particles are still condensed in a single orbital, possibly with a different winding number. This would be the case for a strictly quantum-adiabatic process, for which the system follows the ground state (GS), namely ${E(t) \sim E_{\text {GS}}(\Phi (t))}$. This would be also the case in the presence of a bath that induces relaxation towards the instantaneous GS. But such scenarios are not realistic because they require extremely slow sweep, and because we would not like to expose the system to external dissipation. We therefore ask what would be the result of such protocol for an isolated system that undergoes a realistic slow sweep process. This is precisely the regime where a semiclassical perspective is most effective^49,50,52^. The condensate, which is a many-body coherent state, is represented by a Gaussian-like distribution in phase space. At the preparation stage this *cloud* of points is located at the minimum of the potential. This minimum is a stationary point (SP) of the Hamiltonian. We ask what is the fate of the evolving cloud at the end of the sweep? Is it going to ergodize, or is it going to maintain some coherence? In a larger context we are looking for a theory for the design of QSTP.

**Figure 1:**
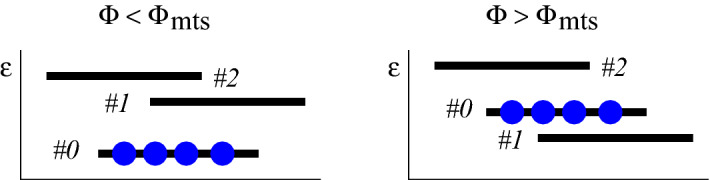
Orbital occupation. For the purpose of illustration we consider a ring with $N=4$ particles. The orbitals are represented by horizontal lines (the horizontal shifts hints the sign of the momentum). Initially (left panel) the particles are condensed in the #0 momentum orbital. As $\Phi$ is increased beyond $\Phi _{\text {mts}}$ this configuration becomes metastable (right). We ask what is the moment when the #0 orbital is depleted, and what is the final distribution of the particles. The $N=4$ system has 15 energy levels that corresponds to the different possibilities to distribute the particles between the orbitals. In the presence of non-zero interaction those levels are partially mixed.

**Figure 2:**
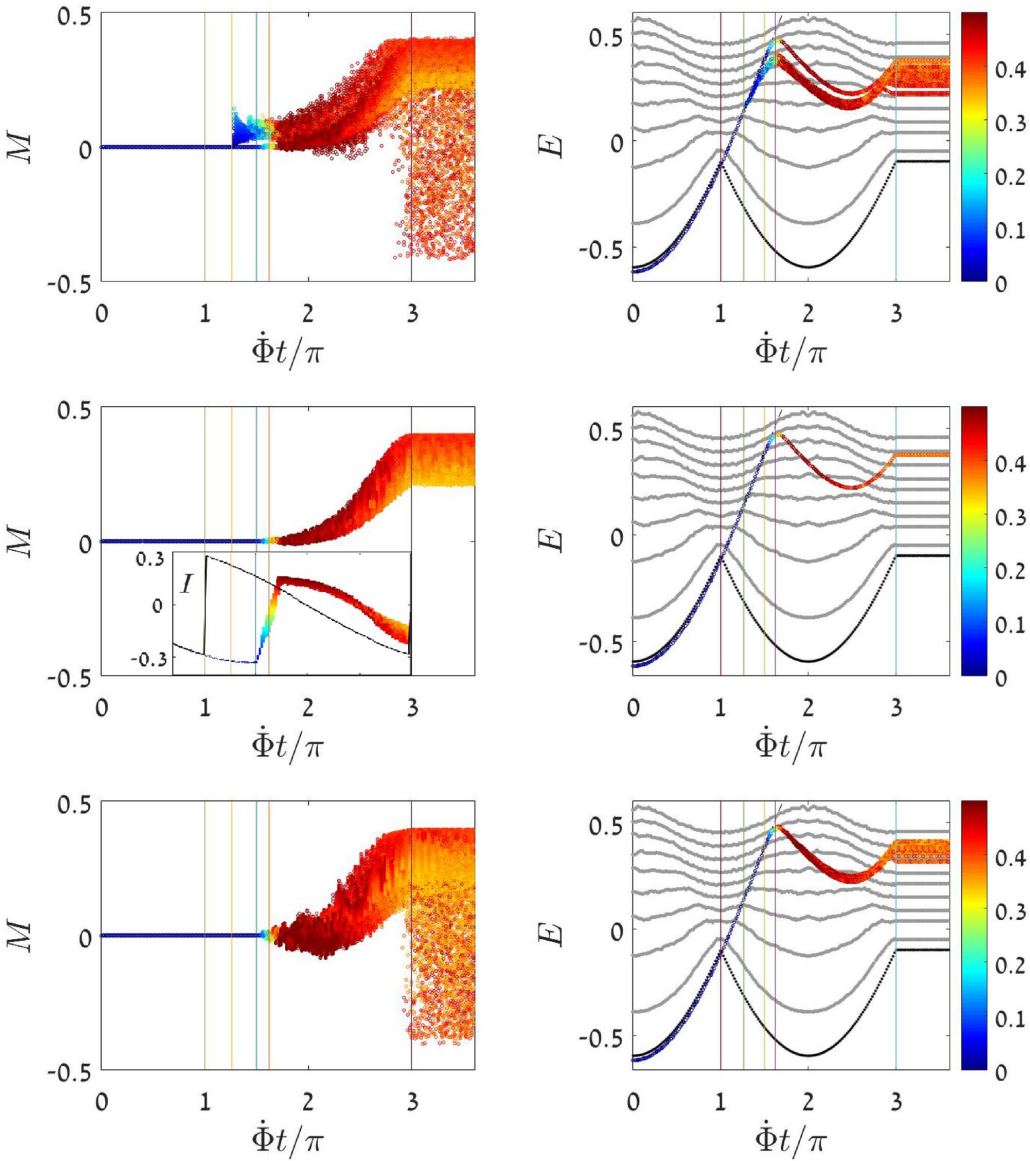
Semiclassical simulation of a sweep process. Here and below we consider a 3-site ring. The initial condensate is represented by a cloud of radius $R = 0.0001$ at $n=0$. **Left**: The (*n*, *M*) coordinates of the evolving trajectories are presented as a function of time. Both coordinates are normalized (${n:=n/N, M:=M/N}$). The *n* values are color coded such that blue corresponds to $n=0$ and red to total depletion. **Right**: The energy *E* of the evolving points as function of time. The dotted line is the ground state energy $E_{\text {GS}}$, and the dashed line is the condensate energy $E_0$. The other lines in the background are subset of adiabatic $E_n$ curves (see text). **Inset** (second row): The current *I* that flows in the ring as a function of time. **Parameters**: The interaction is $u=2.3$, and the associated vertical lines are from left to right $\Phi _{\text {mts}}=\pi$ and $\Phi _{\text {stb}}=1.26\pi$ and $\Phi _{\text {dyn}}=(3/2)\pi$ and $\Phi _{\text {swp}}=1.62\pi$. The units of time have been chosen such that $K=1$. Each row is for a different sweep rate. From up to down we have ${\dot{\Phi }}=3\pi \cdot 10^{-4}$ (slow) and ${\dot{\Phi }}=5\pi \cdot 10^{-4}$ (optimal) and ${\dot{\Phi }}=3\pi \cdot 10^{-3}$ (faster).

### Outline

We present the model Hamiltonian in terms of physically motivated coordinates, and display results of sweep simulations. Then we illuminate our findings by performing step-by step analysis of the energy landscape, and of the phase-space dynamics.

## Results

### The model

We consider a system with *N* bosons in a 3-site ring. The system is described by the Bose–Hubbard Hamiltonian [Methods] with hopping frequency *K* and on-site interaction *U*. The sweep control-parameter is the Sagnac phase $\Phi$, which is proportional to the rotation frequency of the device: it can be regarded as the Aharonov-Bohm flux that is associated with Coriolis field in the rotating frame^59,60^. There are 3 momentum orbitals $k=0,\pm 2\pi /3$. Initially all the particles are condensed in $k=0$. A caricature for the preparation is provided in Fig. 1 (left panel).

Following^51^ we define a depletion coordinate *n* and an imbalance coordinate *M*, such that the occupations of the orbitals are $n_{0}=N{-}2n$, and $n_{\pm }=n{\pm }M$. The model Hamiltonian can be written in terms of (*n*, *M*), and the conjugate phases ${(\varphi ,\phi )}$. Namely [Methods]:

1 $$\begin{aligned} {\mathcal {H}}(\varphi ,n;\phi ,M) = {\mathcal {H}}^{(0)}(\varphi ,n;M) + \left[ {\mathcal {H}}^{(+)} + {\mathcal {H}}^{(-)} \right] \ \ \ \end{aligned}$$

The first term ${\mathcal {H}}^{(0)}$ is an integrable piece of the Hamiltonian that has *M* as a constant of motion:

2 $$\begin{aligned}&{\mathcal {H}}^{(0)}(\varphi ,n;M) \ = \ E_{0} + {\mathcal {E}}_{\parallel } n + {\mathcal {E}}_{\perp } M - \frac{U}{3}M^2 \nonumber \\&+ \frac{2U}{3} (N-2n)\left[ \frac{3}{4}n + \sqrt{n^2-M^2} \cos (\varphi ) \right] \end{aligned}$$

while the additional terms induce resonances that spoil the integrability, and give rise to chaos:

3 $$\begin{aligned} {\mathcal {H}}^{(\pm )} = \frac{2U}{3} \sqrt{(N{-}2n) (n {\pm } M)} (n {\mp } M) \cos \left( \frac{3\phi {\mp } \varphi }{2} \right) \ \ \ \ \end{aligned}$$

The hopping frequency *K* and the Sagnac phase $\Phi$ hide in the expression for the energy of the condensate, and in the detuning parameters:

4 $$\begin{aligned} E_0= & {} -NK \cos \left( \frac{\Phi }{3}\right) + \frac{1}{6}UN^2 \end{aligned}$$

5 $$\begin{aligned} {\mathcal {E}}_{\parallel } \ = \ 3K\cos {\left( \frac{\Phi }{3}\right) } + \frac{1}{6}UN \end{aligned}$$

6 $$\begin{aligned} {\mathcal {E}}_{\perp }= & {} -\sqrt{3}K\sin {\left( \frac{\Phi }{3}\right) } \end{aligned}$$

We also note that the energies of the totally depleted states (${n=(N/2)}$) are

7 $$\begin{aligned} E_{\infty }(M) \ = \ E_0 + {\mathcal {E}}_{\parallel } \frac{N}{2} + {\mathcal {E}}_{\perp } M - \frac{U}{3}M^2 \end{aligned}$$

Note that the latter expression has zero contribution from the ${\mathcal {H}}^{(\pm )}$ terms. The *chaos* affects the pathway between the initial condensate at ${n=M=0}$, and the peripheral depleted states at ${n=(N/2)}$, but has only little effect on the gross features of the energy landscape.

### Metastability

The central point in phase space ${n=M=0}$ is a stationary point (SP) of the Hamiltonian for any $\Phi$, meaning that we have there ${{\dot{n}}=0}$. But this does not mean that this SP is stable. As implied by the caricature of Fig. 1, the condensate at ${n=0}$ is no longer situate at the minimum of the energy landscape once ${ E_0 > \min \{ E_{\infty }(M) \} = E_{\infty }(N/2) }$. This leads to the threshold

8 $$\begin{aligned} \Phi _{\text {mts}} \ \ = \ \ \pi \end{aligned}$$

Once we cross ${\Phi _{\text {mts}}}$ the SP becomes a metastable minimum. Illustrations of the energy landscape for representative values of $\Phi$ can be found in the Supplementary. In the subsequent paragraphs we shall discuss additional thresholds: Once we cross ${\Phi _{\text {stb}}}$ the central SP becomes a saddle in the energy landscape. Once we cross ${\Phi _{\text {dyn}}}$ this saddle becomes dynamically unstable. When ${ E_0 = E_{\infty }(0) }$ a dynamical corridor is opened between the central SP and the peripheral depleted states, leading to the identification of what we call swap transition at ${ \Phi = \Phi _{\text {swp}} }$.

### Semiclassics

The classical (as opposed to semiclassical) treatment of the Hamiltonian is commonly termed Mean Field Theory (MFT). The evolving state is represented by a single point in phase space. We can scale the time such that ${t:= Kt}$, and the occupations such that ${n:= n/N}$. Then one finds that the dynamics is controlled by the dimensionless interaction parameter

9 $$\begin{aligned} u \ \ = \ \ \frac{NU}{K} \end{aligned}$$

Upon quantization (aka “second quantization”) the scaled value of the Planck constant is ${\hbar =1/N}$, see e.g.^49,50,52^. Quantum states can be represented in phase space by their Wigner function. In particular the initial coherent state at $n=0$ is represented in phase-space by a Gaussian-like distribution of radius ${R \sim 1/N }$.

What we call “semiclassical treatment” is far better and reliable compared to MFT, and is commonly called Truncated Wigner Approximation (TWA). Within the framework of TWA the Moyal brackets are approximated by Poisson brackets, which means that the Wigner function is propagated by the classical equations of motion.

The TWA is very accurate as long as quantum tunneling is neglected. The tunneling amplitude scales as $\exp [-\text {Action}/\hbar ]$, where ${\hbar =1/N}$. Therefore it is much slower compared with any classical process. Discussion of tunneling in the BHH context can be found in^62^, and later we demonstrate numerically that it can be neglected for a simulation with ${N=30}$ particles.

**Figure 3:**
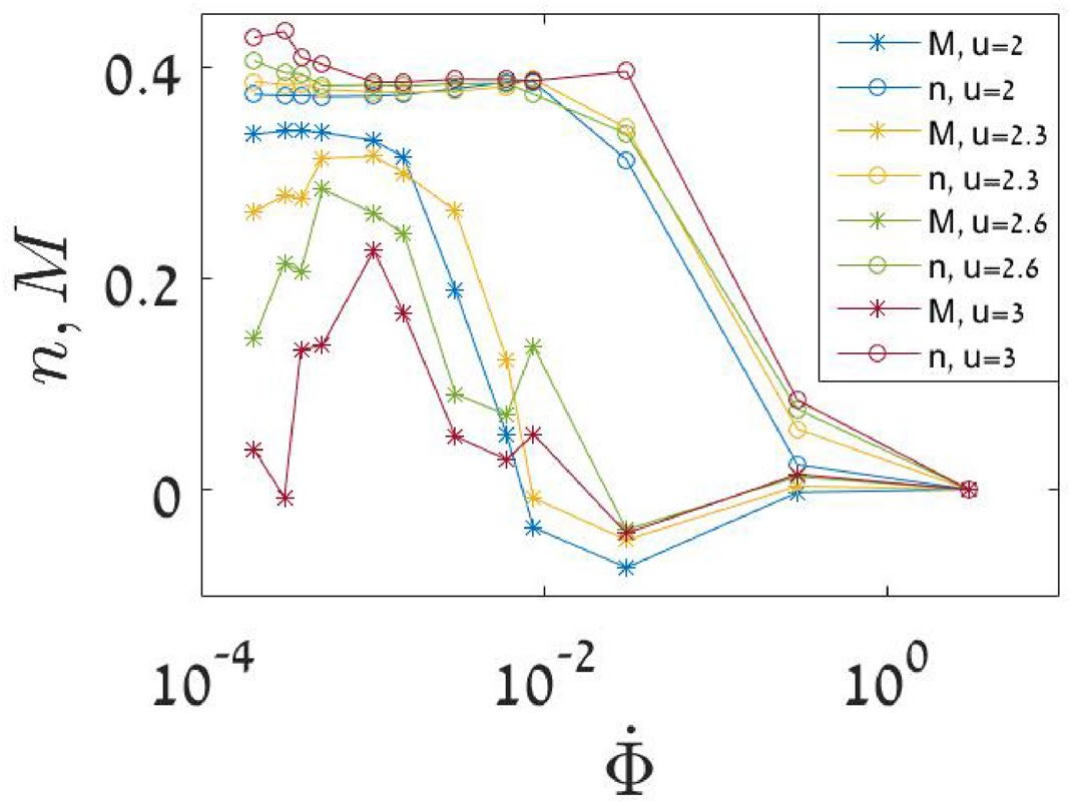
Efficiency of the sweep process. The expectation values $\left\langle n \right\rangle$ and $\left\langle M \right\rangle$ at the end of the sweep process are plotted against ${\dot{\Phi }}$ for misc values of *u*. Note again that the coordinates are normalized (${n:=n/N, M:=M/N}$). The optimal sweep rate is determined by inspection of the maximum of $\left\langle M \right\rangle$, which becomes prominent for large *u*.

### Simulations

We describe the results of semiclassical simulations. Detailed analysis will follow after that. The condensate preparation at $\Phi =0$ is represented by a Gaussian cloud of points in phase space, at the central SP ($n=0$). The evolution of the cloud in a dynamical sweep simulation is demonstrated in Fig. 2. The color-code shows the evolution of the depletion coordinate (*n*), and the vertical position of the cloud points indicate the population imbalance *M* (left panels), or the energy $E={\mathcal {H}}$ (right panels), or the current $I = -{\partial {\mathcal {H}}} / {\partial \Phi }$ as a function of time (inset). For the latter we use the following expression in terms of (*n*, *M*),

10 $$\begin{aligned} I \ = \ \left( n{-}\frac{N}{3}\right) K \sin {\frac{\Phi }{3}} \, + \, \frac{M}{\sqrt{3}} K \cos {\frac{\Phi }{3}} \end{aligned}$$

Note that the cloud is a semiclassical representation of the evolving state. Accordingly, to get the expectation value of the energy or of the current, an average has to be taken over the ensemble of evolving trajectories. In Fig. 2 the average is not taken in order to provide an insight for the dispersion as well.

The cloud follows the ground state energy $E_{\text {GS}}$ only up to $\Phi _{\text {mts}}$. Then it continues to follow the condensate energy $E_0$ during an additional time interval. The cloud *starts* spreading not before $\Phi _{\text {stb}}$, and not later than $\Phi _{\text {dyn}}$. The spreading is indicated by the departure of energy from $E_0$. The depletion of the condensate is indicated by the color that changes abruptly from blue ($n=0$) to red ($n{\sim }N/2$). It takes place during a distinct short time interval when $\Phi (t) \sim \Phi _{\text {swp}}$. The depletion stage is also clearly reflected as a jump in the current-versus-time plot. Finally, the subsequent evolution after the depletion does not follow any of the adiabatic $E_n$ curves, as discussed further below.

We display in Fig. 2 three representative simulations: very slow sweep (top row), *optimal* sweep rate (middle row), and faster sweep (lower row). The results for many such simulations are gathered in Fig. 3, where the dependence of $\left\langle n \right\rangle$ and $\left\langle M \right\rangle$ on the sweep rate is demonstrated for different values of the interaction *u*. What we call optimal sweep rate provides the most coherent outcome (minimum dispersion). Contrary to the traditional dogma, it is not true that “slower is better”.

### Adiabatic evolution

**Figure 4:**
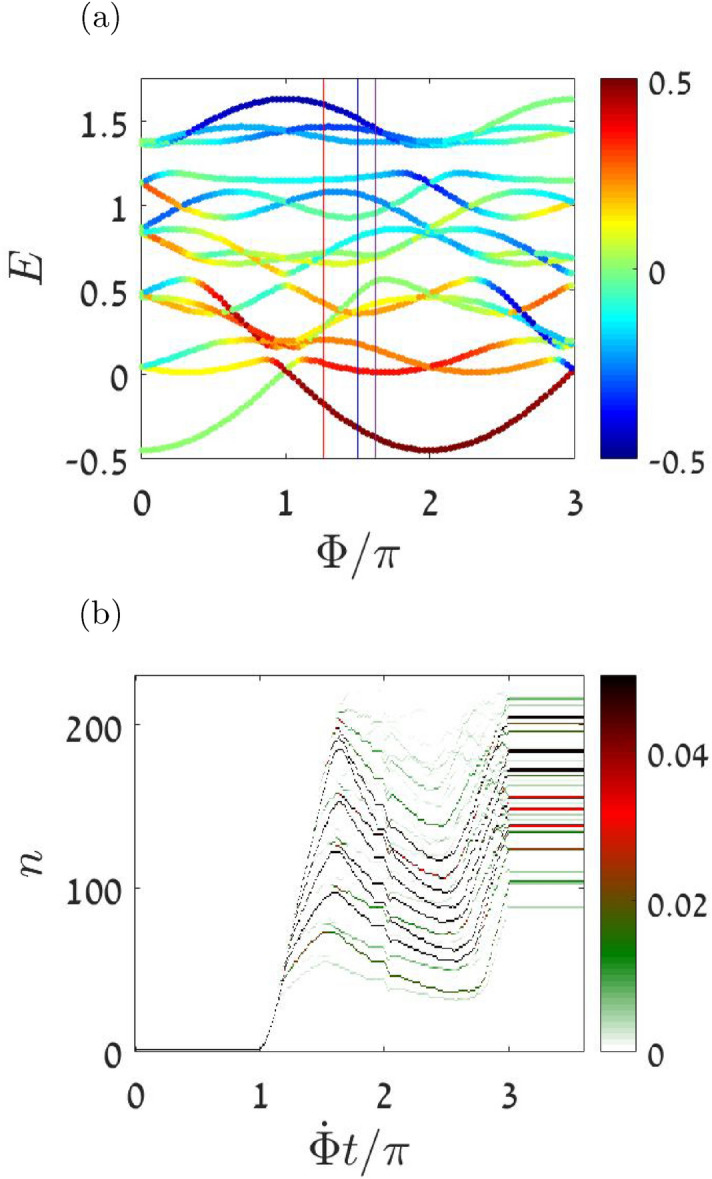
Quantum spreading in few particle system. Even for small number of particles the semiclassical perspective is useful. **Upper panel:** The many body energy levels $E_n$ for $N=4$ particles in a 3-site ring as a function of $\Phi$ for $u=2.3$. The points are color-coded by the expectation value of *M*. **Lower panel:** The quantum evolution of $N=30$ particle ring is imaged. Each row is the color-coded probability ${p_n=|\left\langle E_n \big | \psi (t) \right\rangle |^2}$ as a function of time. For larger *N* we expect a very good quantitative correspondence with the semiclassical simulations of Fig. 2.

It is illuminating to discuss the $\Phi$ dependence of the energy landscape using a quantum “energy level” language. The parametric evolution of the many body eigen-energies is presented in Fig. 4a. If the system were completely chaotic, then we could associate each $E_n$ with a micro-canonical energy surface that encloses a phase space volume

11 $$\begin{aligned} n \ \ = \ \ {\mathcal {N}}(E) \ \ \ \text {[Planck cells]}. \end{aligned}$$

Here, for a given number of particles, we have a system with $d=2$ degrees of freedom, and ${\mathcal {N}}(E)$ is the 2*d* hyper-volume of ${{\mathcal {H}}<E}$ divided by $(2\pi \hbar )^d$. Irrespective of chaos, a practical numerical procedure to find the phase-space volume is to invert the dependence $E=E_n$ where ${n=1,2,3\ldots }$. The validity of this statement is implied by the Wigner-Wyle formalism. The representative $E_n$ curves in the background of Fig. 2 have been calculated using this procedure with $N=30$.

For an adiabatic sweep, the phase-space volume Eq. (11) is the so-called adiabatic invariant^2–4^. This statement assumes a globally chaotic energy surface. In the classical context we say that during an adiabatic sweep the system stays in the same adiabatic energy surface. In the quantum context we say that the system stays in the same adiabatic energy level.

In a strictly quantum-adiabatic scenario, the system stays in its ground state with energy $E_{\text {GS}}(\Phi )$, and therefore the population is fully depleted from $k=0$ to the other orbitals. Such quantum adiabaticity cannot be observed for a realistic sweep rate, because it requires many-body tunneling from a metastable minimum of the energy landscape^62^. Consequently, for large *N*, the semiclassical picture provides a sound approximation. In Fig. 4b we demonstrate that even a circuit with small number of particles ($N=30$) follows a semiclassical-like scenario.

The semiclassical adiabatic scenario excludes the possibility of tunneling, and therefore can start only when ${\Phi (t) > \Phi _{\text {stb}}}$, namely, once the central SP becomes a saddle in the energy landscape. In order to determined $\Phi _{\text {stb}}$ we use the Bogolyubov procedure, which brings the Hamiltonian in the vicinity of the SP to a diagonalized form:

12 $$\begin{aligned} {\mathcal {H}} \ \ \approx \ \ E_0 + \sum _q \omega _q c_q^{\dagger } c_q \end{aligned}$$

Explicit results for the Bogolyubov frequencies are provided in the Methods section. The SP becomes a saddle once the $\omega _q$ do not have the same sign. This happens for $\Phi$ larger than

13 $$\begin{aligned} \Phi _{\text {stb}} = \ \ 3 \arccos {\left( \frac{1}{6} \left( \sqrt{u^2+9}-u\right) \right) } \end{aligned}$$

The topography at the vicinity of the central SP, once it becomes a saddle is as follows: it is still a minimum in the $M=0$ subspace, while away from $M=0$ the energy floor is lower (see the Supplementary for plots of the (*M*, *E*) energy landscape). Nevertheless, we see from the simulation of Fig. 2 that spreading away from the central SP starts only at a later stage, whereafter the cloud departs the $E_0$ curve, neither follows any of the $E_n$ curves.

### Quench-related spreading

Let us consider first the simpler scenario of preparing a cloud at ${n=0}$, which is the $\Phi =0$ ground state, and then evolving it with ${\mathcal {H}}(\Phi \ne 0)$, aka a quench process. The SP for ${t>0}$ (after the quench) is dynamically unstable if the Bogolyubov frequencies become complex. This happens (see Methods) for $\Phi$ larger than

14 $$\begin{aligned} \Phi _{\text {dyn}} \ \ = \ \ \frac{3}{2}\pi \end{aligned}$$

**Figure 5:**
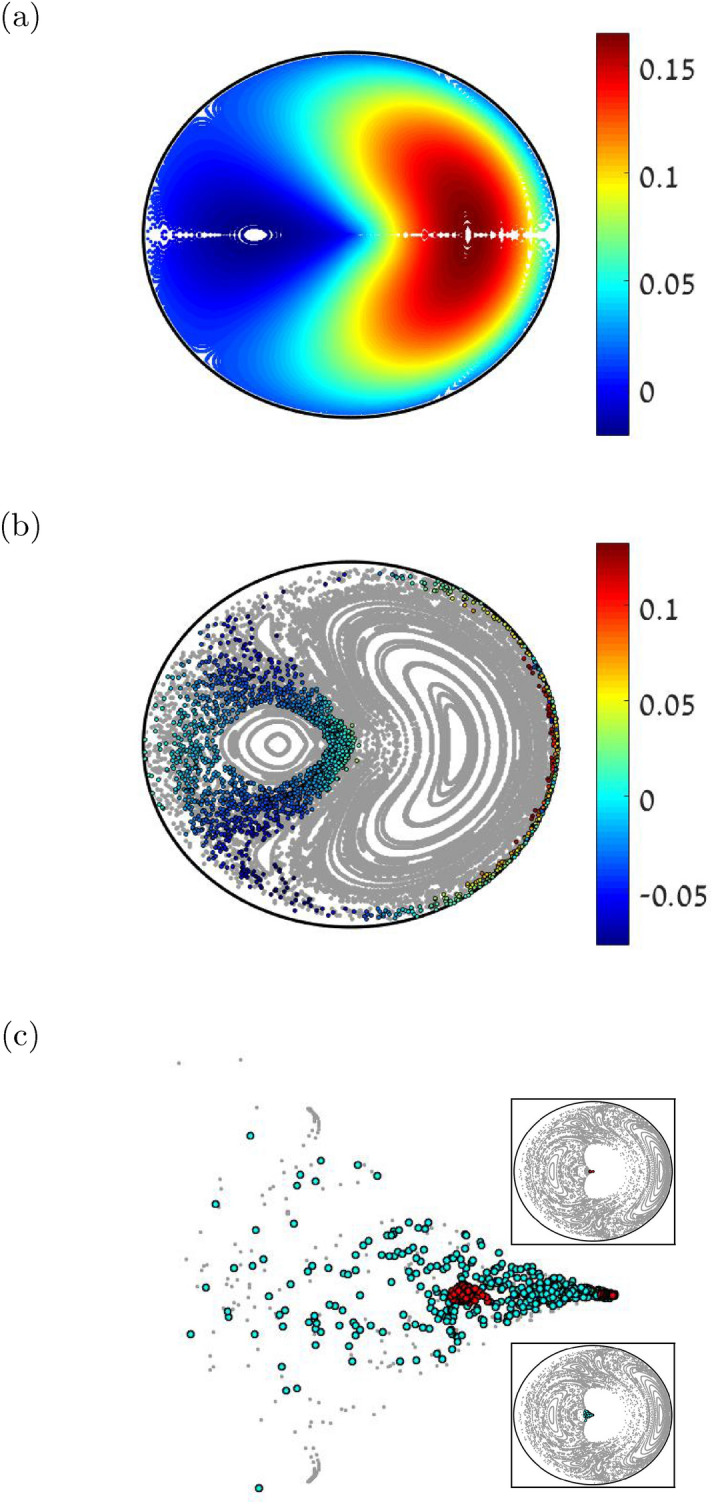
Spreading and depletion. (**a**) Image of *M* for the phase space points of the energy surface ${\mathcal {H}}^{(0)}(\varphi ,n;M)=E_0$. The interaction is $u=2.3$ and ${\Phi =1.61\pi \sim \Phi _{\text {swp}}}$. (**b**) Poincare section for the same $\Phi$ at the same energy (gray trajectories), and a spreading cloud (colored trajectories) following a *quench* to this $\Phi$ value. The initial cloud is the preparation at ${n=0}$. It spreads away from the central SP, and stretches along the chaotic corridor. Its points are color-coded by *M*. (**c**) The spreading cloud in the *sweep* simulation. Upper inset (red points): The sweep rate is ${{\dot{\Phi }}=3\pi \cdot 10^{-4}}$ (slow). The snapshot is taken at ${\Phi \sim \Phi _{\text {stb}}}$. An inner piece of the cloud is still locked in the tiny $n=0$ stability island, and therefore has energy close to $E_0$. An outer piece of the cloud was formed due to very slow spreading in the chaotic corridor, and therefore has lower energy. The Poincare section at the background is adjusted to this lower energy. Lower inset (blue points): The further evolution of the same cloud after we stop the sweep at ${\Phi =\Phi _{\text {stb}}}$ and wait to see further ergodization in the chaotic strip. The upper inset would look like that if the sweep were much slower. Main panel: Zoom that displays the red and the blue clouds of the insets.

After the quench the cloud spreads away from $n=0$ in the landscape that is described by Fig. 5a, as illustrated in Fig. 5b. The Poincare section there shows that the stability island is taken-over by a chaotic strip. The points of the spreading cloud are colored. The other trajectories, that do not belong to the cloud, are not color-coded. If they were color-coded, one would see that for quasi-regular trajectories *M* is approximately a constant of motion.

**Figure 6:**
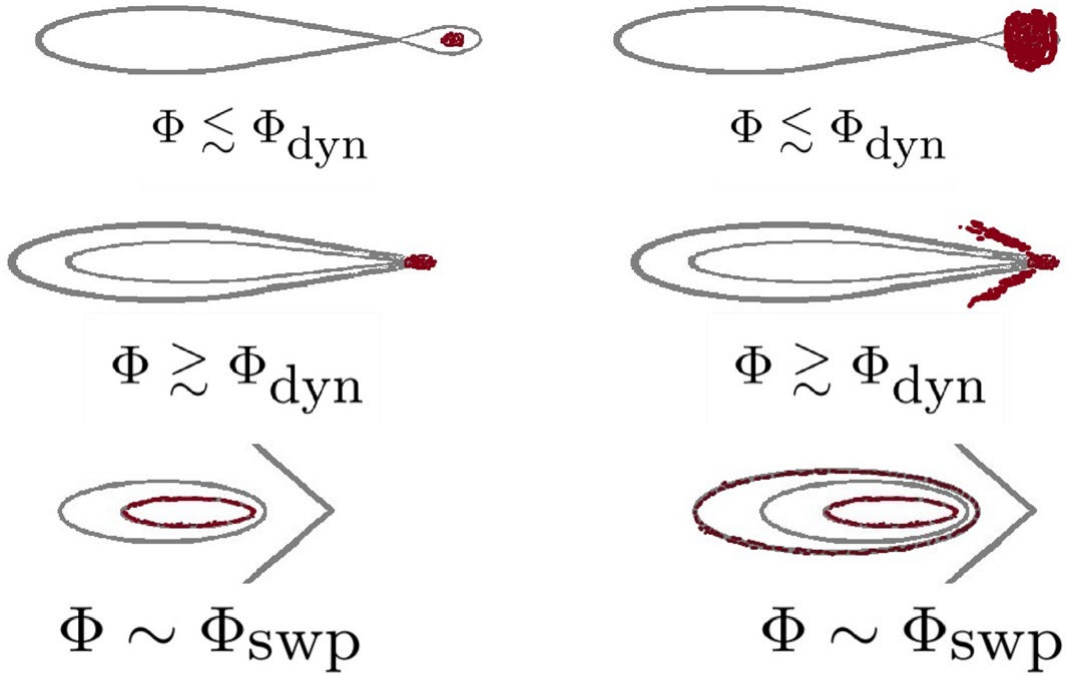
The spreading mechanism. The dynamics of Fig. 5 is caricatured for an optimal sweep (left panels) and for a slow sweep (right panels). The panels are ordered by time (from top to bottom). For an optimal sweep, chaos has no time to induce spreading, therefore, even if the cloud is larger (not displayed) the spreading process looks the same. For a slow sweep the outer part of the cloud has the time to spread way from the center along the chaotic strip. This chaotic spreading is initiated in the range ${ [\Phi _{\text {stb}}, \Phi _{\text {dyn}}] }$, while the former takes place after ${\Phi _{\text {dyn}} }$, as clearly observed in the upper left panel of Fig. 2.

**Figure 7:**
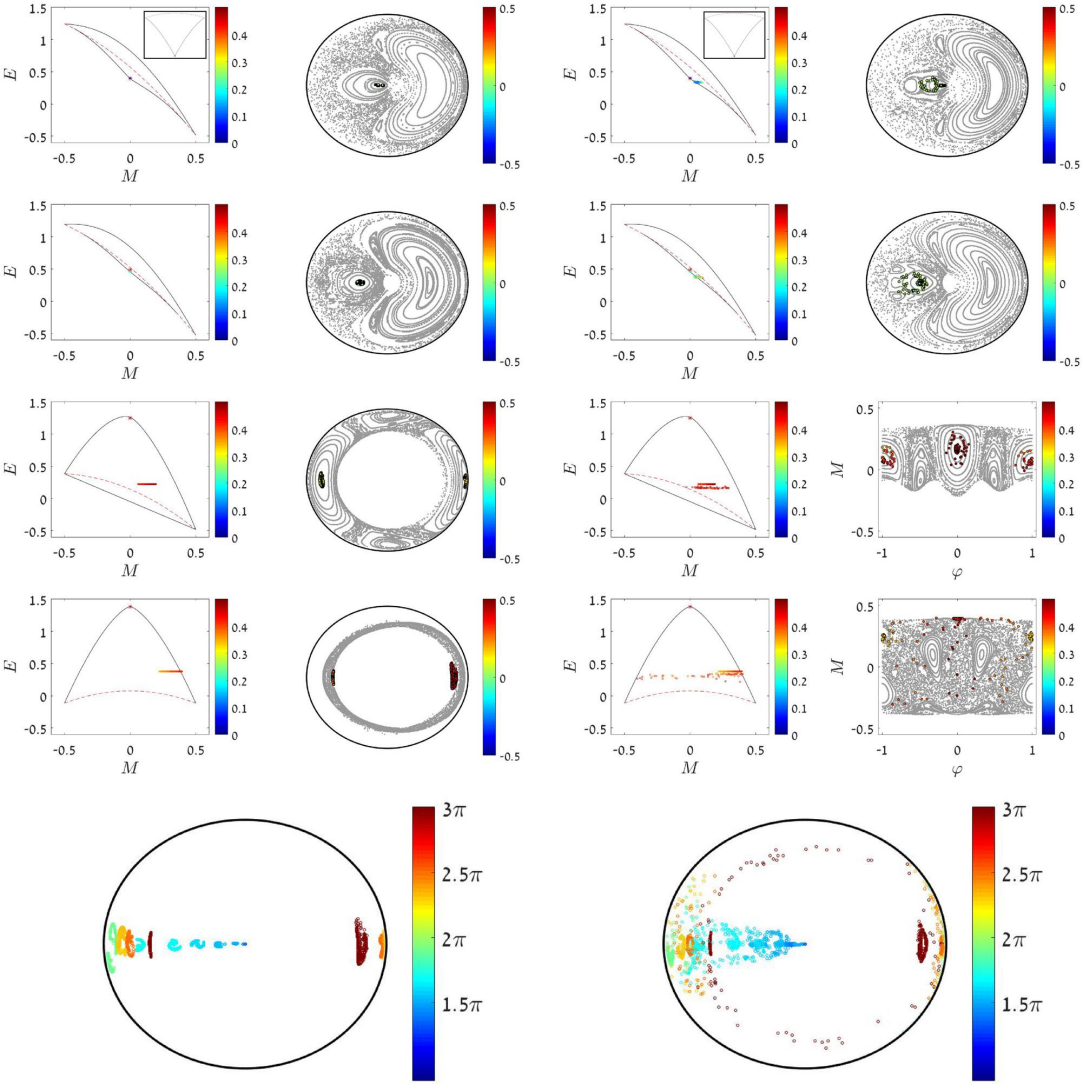
Phase space perspective for the simulations of a sweep process. The rates are ${\dot{\Phi }}=5\pi \cdot 10^{-4}$ (left set of panels) and ${\dot{\Phi }}=3\pi \cdot 10^{-4}$ (right set of panels). The interaction is $u=2.3$. The initial preparation is a condensate at $n=0$ (represented by a red star). Initially it is the minimum of the energy landscape (see insets). Snapshots are taken after $\Phi _{\text {dyn}}$ is crossed, at $\Phi =1.51\pi ,1.6\pi ,2.5\pi ,3\pi$, where the central SP in no longer a local minimum, and furthermore it is dynamically unstable. Consequently the cloud is free to spread away from ${n=M=0}$. *First column of each set:* snapshots of the evolving cloud in (*E*, *M*) space, where the points are color-coded by *n*. *Second column of each set:* The cloud points, color-coded by *M*, are overlayed on the $(\varphi ,n)$ Poincare section. Two panels use non-polar $(\varphi ,M)$ coordinates for enhanced resolution. *Bottom of each set:* the evolving cloud in $(\varphi ,n)$ Poincare coordinates. Snapshots of the cloud are taken at different moments, and are color-coded by $\Phi$. Blue is the initial cloud, and red is its final distribution.

### Sweep-related spreading

We now consider again a quasi-static sweep process. Naively, we might expect that spreading will start once $\Phi _{\text {dyn}}$ is crossed. But a more careful inspections reveals that the QS limit is subtle. We see from the upper left panel of Fig. 2, and from Fig. 5c that for a slow sweep the cloud splits into two pieces. The dynamics is caricatured in Fig. 6. The reason for the splitting is related to the co-existence of two different mechanisms. One resembles the quench scenario. Namely, somewhere in the range ${ [\Phi _{\text {stb}}, \Phi _{\text {dyn}}] }$ spreading is initiated along the chaotic strip. But a different spreading mechanisms comes into play after $\Phi _{\text {dyn}}$ is crossed. This second mechanism dominates the “optimal sweep” of Fig. 2. For an optimal sweep the chaos-related spreading mechanism has no time to develop.

The additional sweep-related mechanism is not related to chaos, but to the bifurcation of the stability island. It obeys the Kruskal–Neishtadt–Henrard theorem^13–23^, namely, the cloud is drained into the emerging stability island. The full optimal sweep scenario is displayed in the left panels of Fig. 7.

### Depletion process

As we already observed in Fig. 2, the spreading of the cloud starts before or latest at $\Phi _{\text {dyn}}$. But looking at the color-code we see that the depletion happens at a distinct moment when $\Phi (t) \sim \Phi _{\text {swp}}$. This is the moment when a corridor connects the central SP $n=0$ with the peripheral region $n=N/2$. In the absence of chaos $n=N/2$ is formally an SP of the ${{\mathcal {H}}^{(0)}(\varphi ,n;M=0)}$ Hamiltonian. Each SP has its own separatrix. For $\Phi = \Phi _{\text {swp}}$ the two SPs have the same energy, and therefore the two separatrices coalesce. From the equation ${ E_0 = E_{\infty }(0) }$ we get

15 $$\begin{aligned} \Phi _{\text {swp}} \ \ = \ \ 3 \arccos \left( -\frac{1}{18}u\right) \end{aligned}$$

Once we add the ${\mathcal {H}}^{\pm }$ terms, this joint separatrix becomes a chaotic strip, what we call “corridor”. The corridor is available for a small range of $\Phi$ around $\Phi \sim \Phi _{\text {swp}}$. During the time interval that the corridor is opened, the central SP is depleted. Both the energy landscape and the evolution are demonstrated in Fig. 7.

### Subsequent evolution

We already pointed out that strict classical adiabaticity in the QS sense of Kubo does not hold for our scenario: for ${\Phi (t) > \Phi _{\text {swp}}}$ the system does not follow any of the $E_n$ curves. The reason for that is figured out by further inspection of the dynamics. For ${\Phi (t) > \Phi _{\text {swp}}}$ the chaotic strip decomposes into quasi regular tori. Consequently a different adiabatic scenario takes over, that of Einstein and Landau, where adiabatic invariants are the “actions” of the tori. Each piece of the cloud is locked in a different torus, and therefore we do not observe in Fig. 7 further ergodization in the *M* direction.

### Quasi static average

Without any approximation we always have ${{\dot{E}} = -\left\langle I \right\rangle _t {\dot{\Phi }}}$. In the Ott-Wilkinson-Kubo formulation of linear response theory^2–8^, it is assumed that for a QS process the instantaneous average can be replaced by an evolving microcanonical average $\left\langle I \right\rangle _{E}$ due to quasi-ergodicity. But we are not dealing with a globally chaotic energy surface. Rather, the cloud occupies at any moment only a fraction of the energy shall, or a set tori that depart from the microcanonical shell. We use the notation $\left\langle I \right\rangle _{QS}$ for the corresponding average. Accordingly

16 $$\begin{aligned} dE \ \ = - \left\langle I \right\rangle _{QS} \ d\Phi \end{aligned}$$

For a system with 2 freedoms the QS average is well defined: at any moment the ergodic region that is accessible for the evolving cloud is bounded by KAM surfaces. This is not true if we had more than 2 freedoms: then the accessible region would likely exhibit a more complicated dependence on the rate of the sweep. Anyway, in the present context the current of Eq. (10) reflects the occupation of the orbitals, and therefore can be expressed in terms of (*M*, *n*). The expectation value of the current can be calculated for the evolving cloud of the simulation, see inset of Fig. 2, and we have verified numerically (not shown) that it agrees with Eq. (16).

### Post-sweep ergodization

For a QS process it is expected to witness quasi-ergodic distribution at any moment. For faster sweep the cloud fails to follow the evolving energy landscape, and therefore a post-sweep ergodization stage is expected, as indeed observed in Fig. 2 for the “faster” sweep. But surprisingly post-sweep ergodization stage is also observed if the sweep rate is extremely slow, as observed in Fig. 2 for the “slow” sweep. The reason for that is explained by Fig. 7. Namely, in the case of a very slow dynamics, the cloud is split into several branches as explained previously. Most of it is re-trapped by quasi-integrable tori. But at the very last moment most of the tori are destroyed, and chaos takes-over again. Consequently a fraction of the cloud, that is no longer locked by tori, undergoes post-sweep ergodization.

## Discussion

Disregarding the very well studied 2-site Bosonic Josephosn junction, the trimer is possibly the simplest building block for an atomtronic circuit. It is the smallest ring that possibly can be exploited as a SQUID-type Qubit device^29–32^. The first requirement is to have the possibility to witness a stable superflow^49–51^. The second requirement is to have the possibility to witness coherent operation. The latter is indicated by, say, coherent oscillations between clockwise and anti-clockwise superflow currents^32^. The third requirement is to have the possibility to execute protocols that do not spoil the coherence, meaning that the particles remain condensed in some evolving orbital^61^. In semiclassical perspective it means that an initial Gaussian cloud does not ergodize. One may say that *ergodicity* due to chaos, as opposed to *stability*, is the threat that looms over the condensation of bosons in optical lattices.

Inspired by experiments with toroidal rings^60^, here we considered a lattice ring that undergoes a prototype sweep protocol: increasing $\Phi$ from 0 to $3\pi$ such that the $k=0$ orbital goes from the floor to the ceiling. During this process this orbital is depleted. The details of the process are as follows: As $\Phi$ is increased beyond a value $\Phi _{\text {mts}}$, the followed SP becomes a metastable minimum; For $\Phi$ larger that $\Phi _{\text {stb}}$ it becomes a saddle in the energy landscape of the circuit; Depending on the sweep rate it can maintain dynamical stability up to some larger value $\Phi _{\text {dyn}}$; Beyond this value the SP becomes unstable, but this does not automatically implies that the coherent state is depleted; A fully developed depletion process requires a *corridor* that leads to ergodization within a chaotic sea; Such corridor is opened during a small interval around $\Phi \sim \Phi _{\text {swp}}$; During the chaotic stage of the sweep we witness partial ergodization, and the final state of the system is in general not fully-coherent. An optimal sweep rate can be determined.

In a larger perspective we emphasize that the traditional view of adiabaticity is not enough in order to a address a QSTP for a system that has *mixed* integrable and chaotic dynamics. Some historical background is essential in order to appreciate this statement. On the one extreme we have the *Einstein-Landau theory* for adiabaticity for integrable systems^1^. On the other extreme we have the *Kubo-Ott-Wilkinson picture* of adiabaticity in chaotic systems^2–8^, which is associated with energy absorption in accordance with linear-response theory. But realistic systems are neither integrable nor chaotic, but rather have mixed phase space whose topological structure changes during the sweep process. The simplest scenario is separatrix crossing, that can be addressed using the Kruskal–Neishtadt–Henrard theorem^13–23^. More generally tori can merge into chaos, and new sets of tori can be formed later on. This leads to anomalous dissipation^9,10^ and irreversibility in the QS limit^11,12^. With the same spirit we have explored in this work the mechanisms that are involved in QS *transfer* protocols, and also the non-trivial dependence of the outcome on the sweep rate.

## Methods

### The Hamiltonian

The BHH for an *L*-site rotating ring is

17 $$\begin{aligned} {\mathcal {H}} = \sum _{j=1}^{L} \left[ \frac{U}{2} {a}_{j}^{\dagger} {a}_{j}^{\dagger} {a}_{j} {a}_{j} - \frac{K}{2} \left( {e}^{i(\Phi /L)} {a}_{j{+}1}^{\dagger} {a}_{j} + \text {h.c.} \right) \right] \ \ \ \end{aligned}$$

where *j* mod(*L*) labels the sites of the ring, the *a*-s are the bosonic field operators, and $\Phi$ is the Sagnac phase.

It is convenient to switch to momentum representation. For a clean ring the momentum orbitals have wavenumbers $k=(2\pi /L)\times \text {integer}$. One defines annihilation and creation operators ${b}_{k}$ and ${b}_{k}^{\dagger }$, such that $b_k^{\dagger } = \frac{1}{ \sqrt{L} } \sum _j \mathrm {e}^{ikj} a_j^{\dagger }$ creates bosons in the *k*-th momentum orbitals. Consequently the BHH takes the form

18 $$\begin{aligned} {\mathcal {H}} \ \ = \ \ \sum _{k} \epsilon _k b_k^{\dagger }b_k \ + \ \frac{U}{2L} \sum ' b_{k_4}^{\dagger }b_{k_3}^{\dagger }b_{k_2}b_{k_1} \end{aligned}$$

where the constraint ${k_1{+}k_2{+}k_3{+}k_4=0}$ mod($2\pi$) is indicate by the prime, and the single particle energies are

19 $$\begin{aligned} \epsilon _k \ = \ -K \cos \left( k- \frac{\Phi }{L} \right) \end{aligned}$$

Later we assume, without loss of generality, that the particles are initially condensed in the ${k=0}$ orbital. This is not necessarily the ground-state orbital, because we keep $\Phi$ as a free parameter. Note that we optionally use *k* as a dummy index to label the momentum orbitals.

### Trimer hamiltonian

For the purpose of semiclassical treatment we express the Hamiltonian in terms of occupations and conjugate phases. For the 3-site ring ($L=3$) we get:

20 $$\begin{aligned} {\mathcal {H}}= & {} \sum _{k=0,1,2} \epsilon _k n_k \ + \ \frac{U}{6} \sum _k n_k^2 \ + \ \frac{U}{3} \sum _{k'\ne k} n_{k'} n_{k} \nonumber \\&+ \frac{U}{3} \sum _{k'' \ne k' \ne k} \left[ n_{k'}n_{k''}\right] ^{1/2} \ n_{k} \ \cos \left( \varphi _{k''} + \varphi _{k'} - 2 \varphi _{k} \right) \end{aligned}$$

We define ${q_1 = \varphi _1 - \varphi _0 }$ and ${q_2 = \varphi _2 - \varphi _0 }$ where the subscripts refers to ${k_{1,2}=\pm (2\pi /3)}$. Using the notation

21 $$\begin{aligned} {\mathcal {E}}_k \ = \ (\epsilon _{k}-\epsilon _{0}) + (1/3)NU \end{aligned}$$

we get ${{\mathcal {H}} = {\mathcal {H}}^{(0)} + \left[ {\mathcal {H}}^{(+)} + {\mathcal {H}}^{(-)} \right] }$ with

22 $$\begin{aligned} {\mathcal {H}}^{(0)}= & {} \ \epsilon _{0}N + \frac{U}{6}N^2 \ + \ {\mathcal {E}}_1 n_1 + {\mathcal {E}}_2 n_2 \nonumber \\&- \ \frac{U}{3} \left[ n_1^2 + n_2^2 + n_1n_2 \right] \nonumber \\&+ \ \frac{2U}{3} (N{-}n_1{-}n_2) \sqrt{n_1 n_2} \cos \left( q_1 + q_2\right) \end{aligned}$$

and

23 $$\begin{aligned} {\mathcal {H}}^{(+)} = \frac{2U}{3} \sqrt{(N{-}n_1{-}n_2) n_1} \ n_2 \cos \left( q_1 - 2 q_2 \right) \ \ \ \ \end{aligned}$$

while ${\mathcal {H}}^{(-)}$ is obtained by swapping the indices (${1 \leftrightarrow 2}$).

### Compact form

It is more convenient to use the coordinates

24 $$\begin{aligned} \phi [\text {mod}(4\pi )] \ = & \ \ q_1-q_2 \ = \ \varphi _1 - \varphi _2 \nonumber \\ \varphi [\text {mod}(2\pi )] \ =& \ \ q_1+q_2 \ = \ \varphi _1 + \varphi _2 - 2 \varphi _0 \end{aligned}$$

and the conjugate coordinates

25 $$\begin{aligned} M \ = \ \frac{1}{2}(n_{1} - n_{2}) \ \ \in \left[ -\frac{N}{2},\frac{N}{2}\right] \end{aligned}$$

26 $$\begin{aligned} n \ = \ \frac{1}{2}(n_{1} + n_{2}) \ \ \in \left[ |M|, \frac{N}{2}\right] \end{aligned}$$

Then the Hamiltonian takes the form of Eq. (1) with Eq. (2) and Eq. (3). The energy $E_0$ of the $n=0$ central SP is implied by the first two terms of Eq. (22), leading to Eq. (4). The detuning parameters are

27 $$\begin{aligned} {\mathcal {E}}_{\parallel } \ = \ {\mathcal {E}}_1+{\mathcal {E}}_2 - (1/2)NU \end{aligned}$$

28 $$\begin{aligned} {\mathcal {E}}_{\perp } = \ {\mathcal {E}}_1 - {\mathcal {E}}_2 \end{aligned}$$

leading to Eqs. (5) and (6). Note: if we linearized ${\mathcal {H}}$ with respect to the ${(n_1,n_2)}$ occupations, we would get the Bogolyubov approximation, which is Eq. (2) without the third term ($M^2$), and with ${(N{-}2n)\approx N}$.

### Bogolyubov frequencies

The non-trivial Bogolyubov frequencies in units of $K=1$, see Supplementary, are

29 $$\begin{aligned} \omega _{\pm } = \pm \frac{\sqrt{3}}{2}\sin {\frac{\Phi }{3}} + \sqrt{\left( \frac{3}{2}\cos {\frac{\Phi }{3}}\right) ^2+u\cos {\frac{\Phi }{3}}} \ \ \ \ \ \ \ \end{aligned}$$

**Figure 8:**
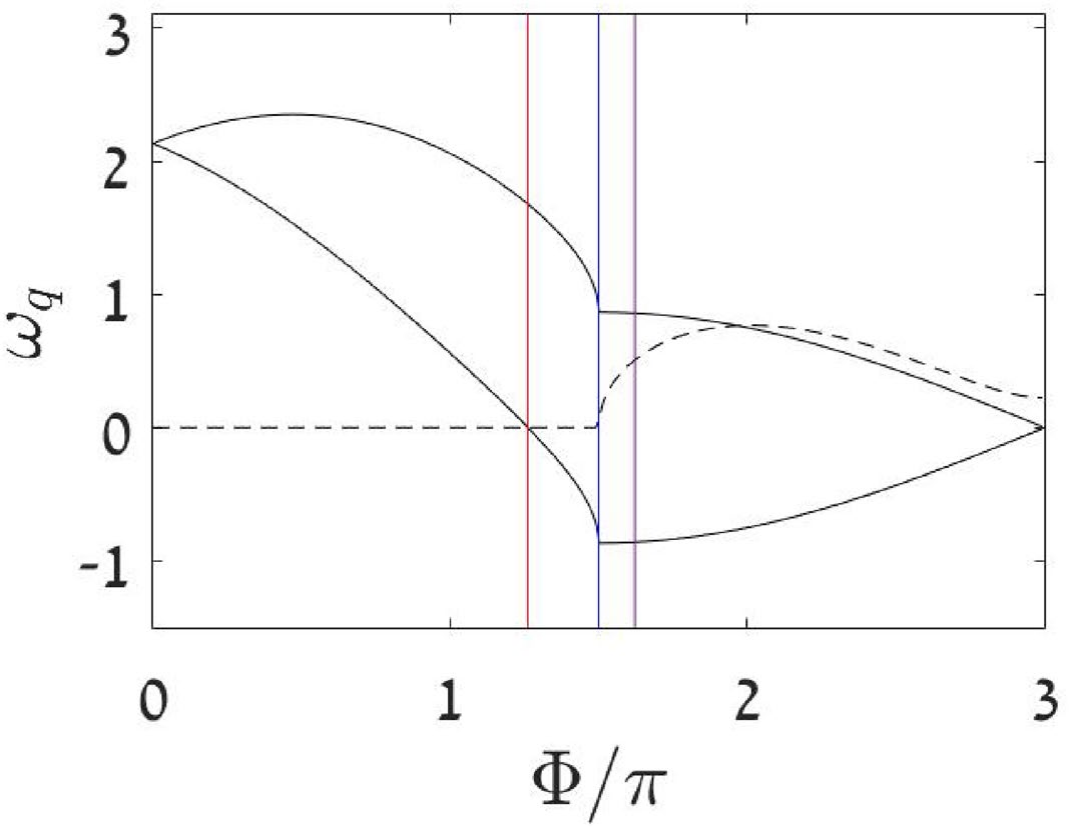
The Bogolyubov frequencies. They are calculated for a ${k=0}$ condensate. The vertical lines from left to right are for $\Phi _{\text {stb}}$, and $\Phi _{\text {dyn}}$, and $\Phi _{\text {swp}}$. The latter cannot be deduced form the Bogolyubov analysis, but requires global understanding of phase space structure.

For positive *u* and $\Phi <\Phi _{\text {stb}}$ the SP is the minimum of the energy landscape, and the Bogolyubov frequencies are positive, see Fig. 8. The SP becomes a saddle once $\omega _{-}$ changes sign and becomes negative. The SP becomes dynamically unstable once the $\omega _{\pm }$ become complex. Note that the energy of the SP, once it becomes unstable, gets above the $M=0$ floor, see Fig. S1 of the Supplementary. By inspection of Eq. (29) we can identify a critical value of the interaction ${u_c=9/4}$. For large interaction (${u>u_c}$) the Bogolyubov frequencies remain complex up to the end of the sweep at ${\Phi =3\pi }$. This indicates that the SP in not at the maximum of the energy landscape, see Fig. S1. The upper most SPs in this region support self-trapped states. For weak interaction (${u<u_c}$) the Bogolyubov frequencies become real and negative once we cross ${\Phi =3\arccos {\left( -(9/4)u\right) }}$, indicating that the SP becomes a stable maximum.

## Supplementary Information

Supplementary Information.
