# Supplementary material for: Quasistatic transfer protocols for atomtronic superfluid circuits
Source: Sci Rep. 2021 Feb 4;11:3136. doi: 10.1038/s41598-021-82386-y (PMC7862411; doi:10.1038/s41598-021-82386-y)
Supplement: Supplementary file 1 — Supplementary Information. [file 41598_2021_82386_MOESM1_ESM.pdf]

Quasistatic transfer protocols for atomtronic superfluid circuits
(Supplementary Material)

Yehoshua Winsten, Doron Cohen
Department of Physics, Ben-Gurion University of the Negev, Beer-Sheva 84105, Israel

[1] Energy landscape

The SPs of the unperturbed Hamiltonian $\mathcal{H}^{(0)}(\varphi, n; M)$ for a given M have to satisfy

$$\frac{\partial \mathcal{H}^{(0)}}{\partial n} = \frac{\partial \mathcal{H}^{(0)}}{\partial \varphi} = 0 \tag{S-1}$$

They are located, for any M, along $\varphi = 0, \pi$, while n should be determined from the equation

$$-\frac{28}{9}n^4 + \left(-4\frac{\mathcal{E}_{\parallel}}{NU} + \frac{14}{9}\right)n^3 + \left(\left(\frac{\mathcal{E}_{\parallel}}{NU} + \frac{1}{2}\right)^2 + \frac{28}{9}\left(\frac{M}{N}\right)^2 - \frac{4}{9}\right)n^2 \\ + \left(4\frac{\mathcal{E}_{\parallel}}{NU} + \frac{2}{9}\right)\left(\frac{M}{N}\right)^2 n - \left(\left(\frac{\mathcal{E}_{\parallel}}{NU} + \frac{1}{2}\right)^2 + \frac{16}{9}\left(\frac{M}{N}\right)^2\right)\left(\frac{M}{N}\right)^2 = 0 \tag{S-2}$$

In the equation above n is the normalized occupation, namely $n := n/N$. This equation has 4 roots, and at most two of them are within the physical range $n \in [0, 1]$. The central SP is $n = 0$ for $M = 0$.

The left column of Fig.S1 illustrates the energy landscape of $\mathcal{H}^{(0)}$ for representative values of Φ. For each M we find the floor (minimum) and the maximum of the energy, and get the Black solid lines that bound the spectrum from below and from above. In particular we indicated by a red point the energy E_0 of the central SP ($n{=}M{=}0$). Note that each point on the upper solid line is formally a peripheral SP ($n = N/2$) of the unperturbed Hamiltonian for a given M, which represents a totally depleted state. Explicit expressions for E_0 and for $E_\infty(M)$ are provide in Eq.(4) and Eq.(7). When the dashed line comes between the solid lines, it means that the peripheral SPs become saddles. This happens in the range

$$3\arccos\left(\frac{1}{3}u\right) < \Phi < 3\arccos\left(-\frac{1}{9}u\right) \tag{S-3}$$

The central SP is the global minimum of the energy landscape up to Φ_{mts} of Eq.(8). It is deduced from the equation $E_0 > E_\infty(N/2)$. For larger Φ the central SP it is still a local minimum, up to Φ_{stb} of Eq.(13). This value can be extracted from the Bogolyubov analysis: the SP becomes a saddle once ω_- of Eq.(29) changes sign and becomes negative. When the red dot comes above the floor, see Fig.S1c, it becomes dynamically unstable, and the Bogolyubov frequencies becomes complex. This happens once we cross Φ_{dyn} of Eq.(14). When the red dot crosses the dashed line (Fig.S1 panels c-d-e), it means that swap of separatrices takes place. The transition happens when $E_0 = E_\infty(M{=}0)$, leading to Φ_{swp} of Eq.(15). At the swap, the two SPs are connected by a single level curve. If the non-integrable terms $\mathcal{H}^\pm$ are included, this level curve becomes a chaotic strip. Thus a corridor is formed, that connects the central SP with the peripheral SPs. This corridor remains open for a small range of Φ values around Φ_{swp}.

For $\Phi{=}3\pi$ the $n{=}0$ central SP gets its highest value, which is not necessarily the maximum of the energy landscape. By the Bogolyubov analysis we can identify a critical value $u_c = 9/4$. For large interaction ($u > u_c$), as in Fig.S1), the central SP is not the maximum of the landscape. Rather, the new maxima support a self-trapped condensates. On the other hand, for weak interaction ($u < u_c$), once we cross

$$\Phi_{\text{dyn-end}} = 3\arccos\left(-\frac{9}{4}u\right) \tag{S-4}$$

the central SP is stable again, and at $\Phi{=}3\pi$ it becomes a stable maximum.

The middle column of Fig.S1 provides vertical section of the energy landscape, namely $E = \mathcal{H}^{(0)}(\varphi, n; M{=}0)$. The right column of Fig.S1 displays Poincare sections at the central SP energy. The trajectories are generated by $\mathcal{H}$ and their section-points are color-coded by M. Note that M is not a constant of motion. Quasi-regular trajectories tend to be mono-chromatic, while chaotic trajectories span a relatively wide range of M values.

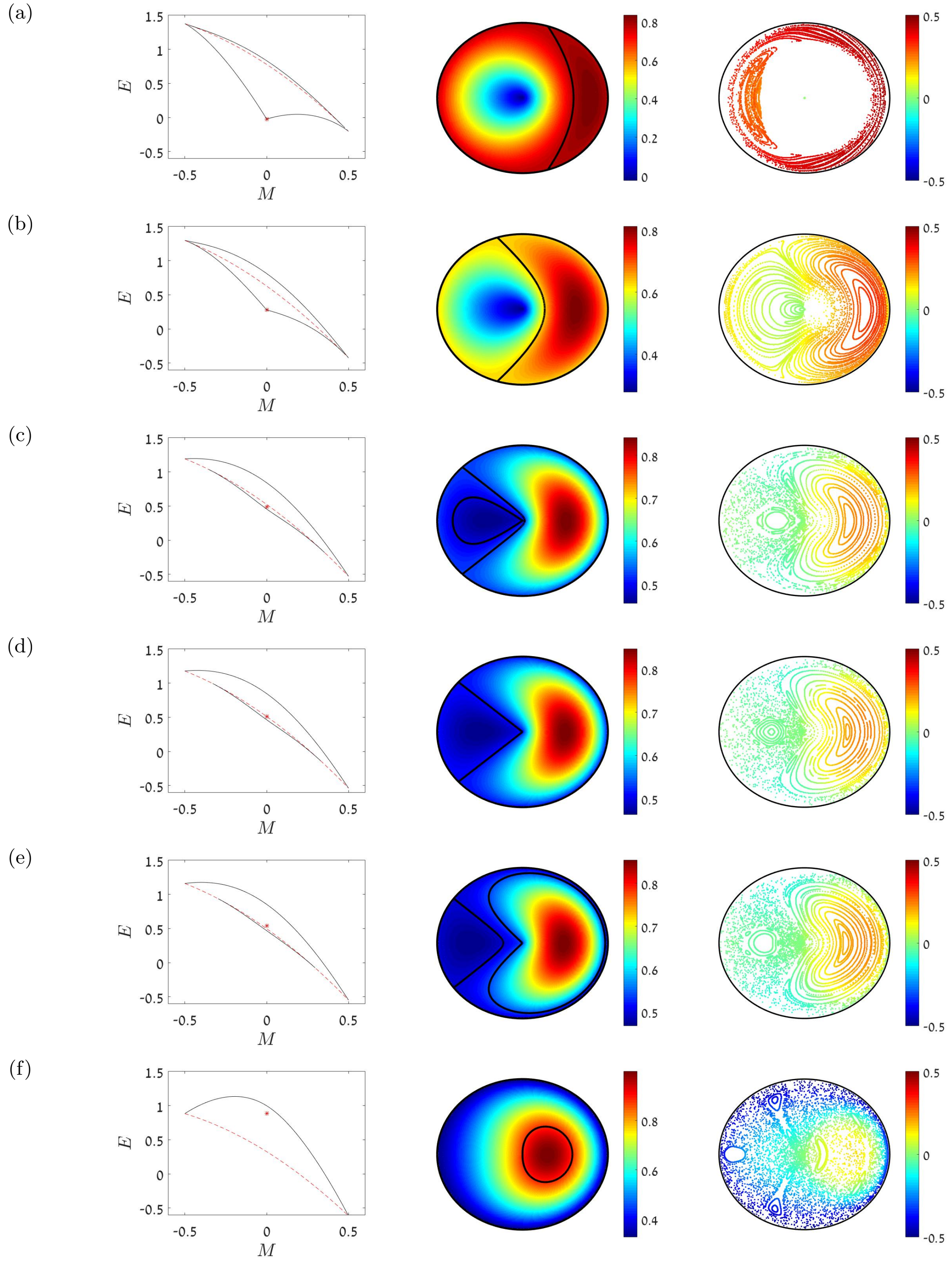

FIG. S1. Left column: the energy landscape of $H^{(0)}$ for $u = 2.3$. Panels (a)-(f) are for $1.1\pi, 1.4\pi, 1.6\pi, \Phi_{\rm swp}, 1.65\pi, 2\pi$. For each M we find the floor (minimum) and the maximum of the energy, and get the Black solid lines that bounds the spectrum from below and from above. We also find for each M the energy of the $n = N/2$ peripheral SP, and get the red dashed line. When the dashed line comes between the solid lines, it means that the peripheral SPs become saddles. The energy of the $n = M = 0$ central SP is indicted by a red dot. When the red dot comes above the floor, it means that the central SP becomes an unstable saddle. When the red dot crosses the dashed line, there is a swap of separatrices. At $\Phi_{\rm swp}$ the two SPs are connected by a single level curve. The middle column provides vertical section of the energy landscape, namely $E = \mathcal{H}^{(0)}(\varphi, n; M{=}0)$. The right column displays Poincare sections at the central SP energy. The trajectories are generated by $\mathcal{H}$ and their section-points are color-coded by M. Note that M is not a constant of motion.

[2] Bogolyubov frequencies

The Bogolyubov procedure brings the Hamiltonian in the vicinity of the SP to a diagonalized form.

$$\mathcal{H} \quad \approx \quad E[\text{SP}] + \sum_q \omega_q c_q^\dagger c_q \tag{S-5}$$

The equations of motion are: $\dot{z} = \mathbb{J}\partial\mathcal{H}$
For one degree-of-freedom the canonical coordinates are $z = (a, \bar{a})$
The symplectic matrix $\mathbb{J}$ is the second Pauli matrix.
Hence an equivalent compact equation is: $\dot{a} = -i\boldsymbol{h}[a, \bar{a}]a$
The SP satisfies $\dot{a} = 0$, provided $\mathcal{H} := \mathcal{H} - \mu N$.
The Hessian (calculated at an SP): $\boldsymbol{H} \equiv \partial\partial\mathcal{H}$
Linearized Hamiltonian: $\mathcal{H} \;\approx\; \frac{1}{2}\sum_{\mu.\nu} \boldsymbol{H}_{\mu,\nu} z_\mu z_\nu$
Linearized equations: $\dot{z} = [\mathbb{J}\boldsymbol{H}]\, z$
Characteristic equation: $\det(\lambda - \mathbb{J}\boldsymbol{H}) = 0$
Eigenvalues are: $\lambda_{q,\pm} = \pm i\omega_q$ (one should be careful about the sign)
One pair of frequencies is zero because the total occupation (N) is conserved.

One site.– Consider one-site Hamiltonian $\mathcal{H} = \epsilon_0 \bar{a}a + \frac{U}{2}\bar{a}\bar{a}aa$
Here $h[a, \bar{a}] = \epsilon_0 + U\bar{a}a$.
The SP for N particles is at $a = \sqrt{N}$ with $\mu = \epsilon_0 + NU$.
Accordingly the hessian at the SP is

$$\boldsymbol{H} = \begin{pmatrix} 0 & \varepsilon_0 - \mu \\ \varepsilon_0 - \mu & 0 \end{pmatrix} + U \begin{pmatrix} \bar{a}\bar{a} & 2\bar{a}a \\ 2\bar{a}a & aa \end{pmatrix}_{a:=\sqrt{N}} \tag{S-6}$$

The characteristic equation gives the trivial frequency $\omega_0 = 0$.

Ring.– For M sites, the zero-momentum SP is associated with $\mu = \varepsilon_0 + (NU/M)$, and we get

$$\boldsymbol{H} = \begin{pmatrix} 0 & \boldsymbol{h}_0 - \mu \\ \boldsymbol{h}_0 - \mu & 0 \end{pmatrix} + \frac{NU}{M} \begin{pmatrix} \mathbf{1} & \mathbf{2} \\ \mathbf{2} & \mathbf{1} \end{pmatrix} \tag{S-7}$$

where $\boldsymbol{h}_0$ is the kinetic part of $\boldsymbol{h}[a, \bar{a}]$ (only hopping terms, no interaction), and $\mathbf{1}$ (identity) and $\mathbf{2}$ (twice the identity) are $M \times M$ diagonal matrices (reflect the interactions). Note that the $\mathbf{2}$ can be absorbed into the kinetic matrix $\boldsymbol{h}_0$, while the $\mathbf{1}$ elements are related to terms of the type $a_j a_j$. Switching to the momentum basis the kinetic matrix becomes diagonal, while

$$\mathbf{1} \mapsto \begin{pmatrix} 1 & 0 & 0 \\ 0 & 0 & 1 \\ 0 & 1 & 0 \end{pmatrix} \tag{S-8}$$

The above matrix includes the $k = 0$ block plus one representative $(k, -k)$ block. If we look on $\mathbb{J}\boldsymbol{H}$, we see that it decouples into blocks. All the block has the structure $\Omega_z \boldsymbol{\sigma}_z + i\Omega_y \boldsymbol{\sigma}_y$, up to a constant. Note that $\Omega_z = \Omega_y$ is an exceptional point with zero eigenvalues. Indeed the $k = 0$ block provides the zero frequencies, and the other blocks (without the $-i$ prefactor) are

$$\begin{pmatrix} \mathcal{E}_k & 0 \\ 0 & -\mathcal{E}_{-k} \end{pmatrix} + \frac{NU}{M} \begin{pmatrix} 0 & 1 \\ -1 & 0 \end{pmatrix} \tag{S-9}$$

where $\mathcal{E}_k = \varepsilon_k - \varepsilon_0 + (NU/M)$. Note that the block with $k \mapsto -k$ provides frequencies with opposite signs. We conclude that

$$\omega_{q,\pm} \;=\; \pm\left(\frac{\mathcal{E}_q - \mathcal{E}_{-q}}{2}\right) + \sqrt{\left(\frac{\mathcal{E}_q + \mathcal{E}_{-q}}{2}\right)^2 - \left(\frac{NU}{M}\right)^2} \tag{S-10}$$

The correctness of the sign convention can be tested by setting $U = 0$.

The Bogolyubov frequencies are calculated as a function of Φ in Fig.S2. The implications of the various crossovers are reflected in the parametric diabatic evolution of the E_0 level in the quantum spectrum (right panels).

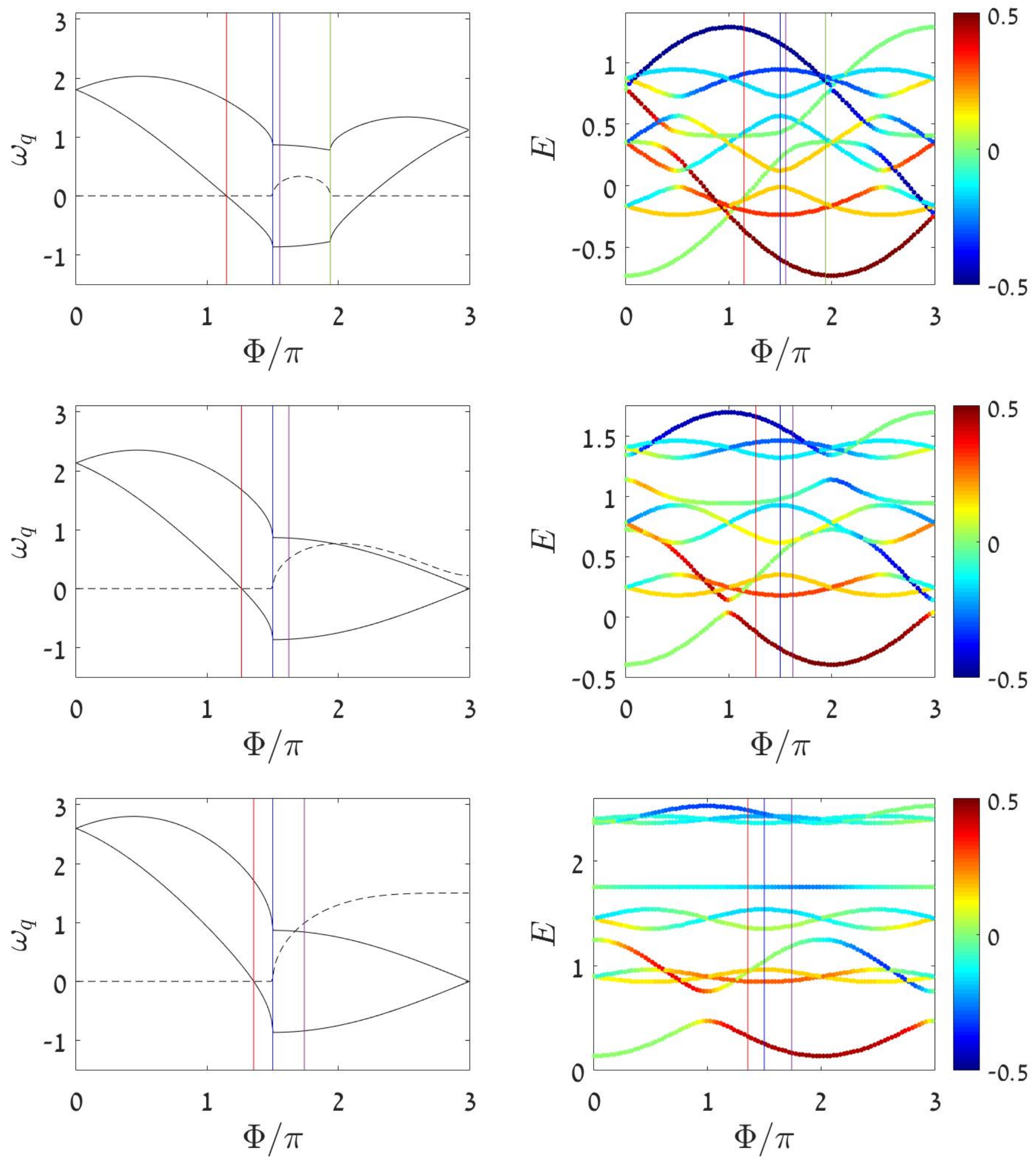

FIG. S2. **Parametric variation of the energy landscape.** Left: The Bogolyubov frequencies for a $k = 0$ condensate. The vertical lines from left to right are for Φ_{stb}, Φ_{dyn} and Φ_{swp}. Right: The many body energy levels E_n for $N = 3$ particles as a function of Φ. The points are color-coded by the expectation value of M. The calculations are done from up to down for $u = 1.0, 2.3, 4.5$. In the first row (weak interaction) also $\Phi_{\mathrm{dyn\text{-}end}}$ is indicated.
